# Pretreatment of waste activated sludge by rotational generator of hydraulic shock

**DOI:** 10.1016/j.ultsonch.2025.107312

**Published:** 2025-03-13

**Authors:** Sabina Kolbl Repinc, Gašper Rak, Blaž Stres, Uroš Novak, Blaž Likozar, Anže Prašnikar, Marko Blagojevič, Benjamin Bizjan

**Affiliations:** a University of Ljubljana, Faculty of Civil and Geodetic Engineering, Jamova 2, 1000 Ljubljana, Slovenia; b National Institute of Chemistry, Hajdrihova 19, 1000 Ljubljana, Slovenia; c University of Ljubljana, Biotechnical Faculty, Jamnikarjeva 101, 1000 Ljubljana, Slovenia; d University of Ljubljana, Faculty of Mechanical Engineering, Aškerčeva cesta 6, 1000 Ljubljana, Slovenia

**Keywords:** Waste activated sludge, Hydraulic shock, Disintegration, Rheology, Methane production, Performance evaluation

## Abstract

This study investigates hydrodynamic performance of a novel sludge pretreatment device based on periodic shock wave generation by a hydraulic hammer mechanism. A falling circular jet of thickened waste activated sludge was repeatedly impacted by a rotating blade, resulting in occurrence of hydraulic shock waves within the liquid region adjacent to the impact. The rotational generator of hydraulic shock (RGHS) treating 10 L of waste activated sludge was operated for 30 liquid passes and at two different rotational speeds producing blade impact velocities of 44 m/s and 70 m/s, respectively. At 70 m/s impact velocity and 30 passes, the device was able to achieve 41.3 % disintegration degree (DD), specific energy consumption (SEC) of 10.4 kWh/kg sCOD released and 9.0 % improvement of produced methane volume over unprocessed sample. Corresponding values for 44 m/s impact regime were DD = 18.7 %, SEC = 8.03 kWh/kg sCOD released and 33.1 % improvement in methane production. In both pretreatment regimes, sludge shear-dependent viscosity was reduced by about 60 %, while physicochemical analysis, FTIR spectra revealed substantial structural changes in WAS, namely median particle size reduction, degradation of proteins and polysaccharides, and microbial cell wall damage, what was notable also on SEM images. Compared to other rotary devices, the novel RGHS can achieve relatively high degree of sludge disintegration while consuming significantly less energy for sludge solubilization, and for methane production enhancement.

## Graphical abstract

**Figure:**
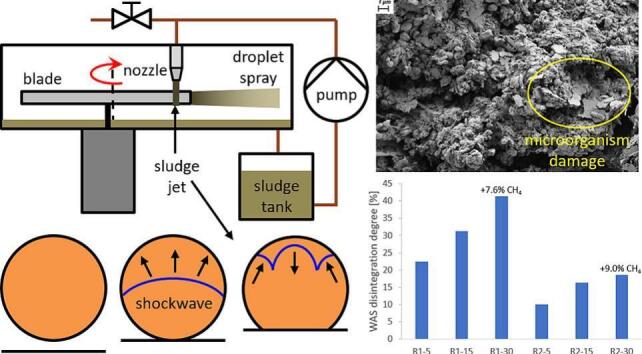

## 1 Introduction

The safety and quality of water resources worldwide are increasingly threatened by growing global population and industrialization, particularly due to the wastewater containing several potentially hazardous contaminants such as industrial chemicals, personal care products and pharmaceuticals, pathogenic microorganisms and microplastics [1]. To minimize water pollution, wastewater (WW) undergoes different treatment processes in wastewater treatment plants (WWTP) before being released into the environment. During WW treatment, large amounts of semisolid residue known as waste activated sludge (WAS) are generated and must be correctly disposed of to avoid soil and water pollution [2]. Most common disposal methods include agricultural reuse and incineration, but the use of these methods is restricted by environmental or economic issues [3]. In the European Union, WAS reuse for crop fertilization is not allowed, thus leaving the costly incineration process (approximately 200 EUR/t of WAS) as one of the few disposal options. To reduce the expense of WAS disposal it is sensible to reduce its amount as much as possible before discarding it from WWTPs. Among the methods for reduction of the amount of discarded WAS, anaerobic digestion (AD) is regarded as a cost-effective and environmentally friendly technology. AD stabilizes sludge, facilitates odor and pathogen removal, and produces methane gas that can be used as a green and renewable fuel also known as biogas [4].

AD is a complex process comprising four main phases, namely hydrolysis, acidogenesis, acetogenesis and methanogenesis, ultimately resulting in methane production and reduction of WAS quantity [2,[5], [6], [7]]. The efficiency of the hydrolysis stage of AD is typically hindered by the presence of high molecular weight organic matter such as complex floc structure (EPS − extracellular polymeric substances) and recalcitrant cell walls [8,9]. The presence of such organic matter increases retention time and required bioreactor size while reducing the biogas yield. However, the efficiency of hydrolysis and subsequent AD phases can be substantially improved by implementation of sludge pretreatment processes prior to the application of AD [10,11]. Pretreatment options include thermal, chemical, biological, sonochemical and hydraulic processes that have been investigated in several studies on laboratory and pilot scale [2,5,8,12].

Each of the pretreatment methods is characterized by certain advantages and shortcomings, with their feasibility depending on WAS degradation effectiveness, scalability for industrial use, operational condition flexibility, and the ease of integration and maintenance in water/wastewater treatment systems [3,13,14]. Compared to other approaches, sonochemical and hydraulic WAS pretreatment has the advantage of relative simplicity and robustness since no added chemicals or microorganisms are involved at this stage. Most common pretreatment methods include hydrodynamic and acoustic cavitation, high pressure homogenization and ball milling [2]. The effectiveness of WAS degradation can be measured by reduction of sludge particle size and structural complexity, increased dewaterability and solubilization, enzyme release and the enhancement of biogas production [2] due to a combined action of shock waves, shear forces, turbulence, and (in the case of cavitation) also chemical effects from collapses of vapor structures that produce localized zones of extremely high pressure and temperature [15,16].

Although numerous studies on the application of mechanical methods for WAS pretreatment has been investigated and demonstrated as effective on the lab scale, many of the above-mentioned methods are of questionable feasibility due to their poor scalability or high energy consumption. Considering that WAS disposal is expensive on its own, only pretreatment methods with high energy efficiency and significant AD enhancement are economically justified. Among mechanical methods, only hydrodynamic cavitation generated by rotating devices has been shown to satisfy both criteria, resulting in significant increase in soluble chemical oxygen demand, dissolved organic matter and methane gas yield [3,13]. In WAS treatment studies, rotational generators of hydrodynamic cavitation (RGHC) employed different rotor and stator geometries, namely dimpled discs [17], serrated discs [1,3,18] pinned discs [1,3,13] and bladed disintegrators [19]. Although cavitation and associated flow phenomena within these devices are highly complex [20,21], the two main mechanisms of WAS degradation are common to all RGHC variants: large amplitude pressure pulsations in form of shock waves, and shear forces. Cavitation of sufficient intensity can also exert chemical effects on sludge through production of reactive chemical species [22], resulting in sludge oxidation that can be both advantageous (reduction of sludge mass) and detrimental (production of chemical substances even more environmentally harmful than WAS itself).

To ensure the maximum effectiveness and repeatability of WAS degradation, the design of AD mechanical pretreatment devices must be optimized to primarily favor hydrodynamic phenomena enhancing the sludge disintegration, while avoiding unproductive energy use (e.g., excessive turbulence or liquid acceleration, too aggressive cavitation generating toxic chemical species…). In designing the pretreatment device for the present study, we have hypothesized that shock waves are by far the most effective means of WAS degradation. A novel rotational generator of hydraulic shock (RGHS) has been considered for this purpose. In its operating principle, the RGHS device mimics rotary impact devices in rain erosion rigs [23,24] and cavitation erosion resistance measurement [25].

**Fig. 1:**
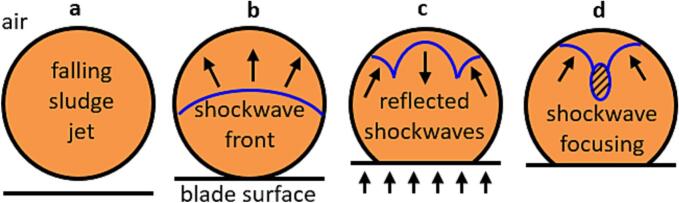
Schematic representation of shockwave generation due to the impact of the blade surface against the sludge jet (cross-section plane normal to initial jet direction). a – unperturbed jet before impact; b – formation of a compression shockwave after impact; c – formation of rarefaction shockwaves after compression shockwave reflection; d – focusing of shock waves (hatched area), possible vapor cavity formation. Arrows mark the direction of blade movement and shockwave propagation.

Compared to HC-based pretreatment devices where shock waves and other WAS degradation mechanisms are primarily driven by cavitation manifesting as rapid implosion of vapor structures, high-speed impacts of the liquid against a solid surface immediately generate hydraulic shock (HS) waves due to the liquid compression and deformation in contact with solid, [26,27] as schematically presented in Fig. 1. Initially unperturbed sludge jet (Fig. 1a) is impacted by the rotating blade (Fig. 1b), producing a positive amplitude shock wave. Initially, the contact edge expands along the blade surface with a velocity greater than that of the shock wave, and the liquid is compressed within the shock envelope developing maximum pressure at the impact surface [28]. The shock wave propagates through the jet until reaching its free surface on the side opposite to the point of impact. Upon reaching the jet surface, the shock wave is reflected towards the blade (Fig. 1c), producing a succession of smaller negative amplitude shock waves that concentrate in a focal point (Fig. 1d). If impact velocity exceeds approximately 100 m/s, the pressure drop in the focal area relative to unperturbed liquid is sufficient to form a non-spheric vapor cavity vigorously collapsing soon afterwards [29], thus entailing hydrodynamic cavitation. The HC formed by such a mechanism facilitates the formation of additional shock waves and high velocity liquid jets in the collapse area [30], potentially enhancing the WAS disintegration effect of the initial HS. Note that Fig. 1 does not depict the later stages of shock- and pressure wave propagation when further reflections from the jet’s free surface and wetted blade surface are possible, but with much lower pressure amplitude thus having only a modest contribution to WAS disintegration.

The performance of our RGHS device has already been investigated in the field of disinfecting virally contaminated water and has been proven superior to Venturi-setup HC [31]. In this study the effects of RGHS shock waves were studied and compared to HC-based devices on WAS degradation in relation to AD enhancement over HC-based devices.

## 2 Experimental set-up

### 2.1 Waste sludge physiochemical parameters

On the day of experiments, 60 L sludge was obtained from Central WWTP Domžale-Kamnik in central Slovenia with size of 149,000 PE (https://www.ccn-domzale.si/index.php/sl/), which treats municipal and industrial wastewater.

The plant also receives septic sludge and sludge from small municipal sewage treatment plants. From these received effluents, first the coarse particles are removed in sieves, then the sludge is pumped to the digesters and the industrial wastewater to the grease trap and the grit chamber. The treatment plant also receives liquid biodegradable waste, which is fed through screens and a shredder into a hygienisation basin, where it is heated at 70 °C for 1 h and then pumped to the anaerobic digesters [32,33].

For this experiment, the activated sludge was collected from a thickener located downstream of the secondary settling tank. The inoculum required for the measurement of the methane potential was obtained from an active anaerobic sludge digester operating in the mesophilic temperature of 38 °C. After the upgrade of the treatment plant in 2017, the hydraulic retention time in the anaerobic reactors is 25 days. The wastewater samples were used within 1 h after collection to prevent any degradation. The characteristics of the WAS and inoculum are given in Table 1.

**Table 1:** Physicochemical parameters of WAS and inoculum.

**Parameter**	**Unit**	**Untreated WAS value ± SD**	**Inoculumvalue ± SD**
pH	−	6.96 ± 0.08	7.29 ± 0.01
EC	μS/cm	1594 ± 26	5217 ± 15
redox	mV	41 ± 5	22.4 ± 0.4
TDS	mg/L	693 ± 4	2.80 ± 0.02
Salinity	‰	0.69 ± 0.01	2.80 ± 0.02
Resistivity	Ωcm	712 ± 4	193 ± 2
TS	% of sample	3.12 ± 0.01	2.30 ± 0.02
VS	% of TS	72.8 ± 0.2	62.4 ± 0.4
COD	mg/L	27450 + 6644	22200 ± 919
sCOD	mg/L	1263 ± 81	−
sTOC	mg/L	631 ± 146	−
sNH_4_-N	mg/L	83 ± 1	993 ± 15
sPO4-P	mg/L	11.1 ± 0.3	−
CST	s	62.3 ± 0.6	−
FOS	mg/L	−	1.6 ± 0.1
TAC	mg/L	−	3.74 ± 0.09
FOS/TAC	−	−	0.43 ± 0.03

### 2.2 Hydraulic shock generator

**Fig. 2:**
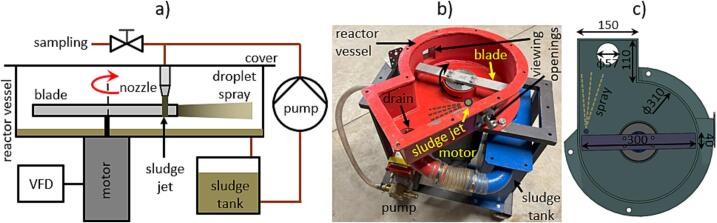
RGHS system used for WAS pretreatment: system scheme (a), photo of partly assembled system without cover (b) and reactor vessel dimensions in [mm] unit (c).

Sludge pretreatment experiments were performed on a rotational generator of hydraulic shock (RGHS) as depicted in Fig. 2. The RGHS comprised a sludge tank, a circulating pump, and a reactor vessel with a rotating blade (Fig. 2a, Fig. 2b). The reactor vessel was volute-shaped with a circular section of 310 mm diameter and a rectangular-shaped extension 110 mm long and 150 mm wide where a sludge drain was installed (Fig. 2c). 10 L of thickened waste activated sludge (WAS) with 3.12 % of dry matter was introduced to the RGHS tank and pumped at a flow rate of 18 L/min into the reactor vessel where the sludge exited vertically through a 12 mm nozzle (15 mm vertical clearance between nozzle outlet and the blade upper edge), forming a falling jet with resulting exit velocity of approximately 2.6 m/s. To ensure a constant flow rate of WAS regardless of the change in its rheological properties due to structural disintegration and temperature increase, a gear pump with stainless steel gears was utilized.

The jet was impacted by a symmetric flat blade (10 mm height, 40 mm width, 300 mm length, 5 mm side wall clearance, 60 mm top clearance, 45 mm bottom clearance) twice per blade revolution at 140 mm radial distance from blade rotational axis. The blade’s rotational speed *f* was controlled by a variable frequency drive (VFD) that supplied power to the electric motor. Upon each impact the falling sludge jet disintegrated into a spray of droplets that was contained by reactor vessel walls and then drained back into the tank. Two viewing openings were installed in the circular side wall section of the reactor vessel, allowing for observation and illumination of the sludge jet impaction process. However, unlike in our previous study where transparent liquid was used [31], high-speed imaging of impacts was not undertaken due to complete opacity of the sludge which prevented the observation of related phenomena within the sludge jet.

The WAS pretreatment experiments were conducted under two different operating conditions, namely at *f* = 3000 RPM and *f* = 4800 RPM, corresponding to 44 m/s and 70 m/s impact velocity, respectively. Respective total power consumption of the device (rotating blade and pump) was 400 W at 3000 RPM and 1150 W at 4800 RPM. Under each condition, 2 L of sludge was sampled at 5, 15 and 30 liquid passes (Np) in addition to a sample of unprocessed WAS. Due to the significant reduction of the remaining sludge volume in the tank after each sampling, the pass duration was adjusted (reduced) accordingly. From the initial temperature of 13.5 °C, the sludge temperature after 30 passes reached 23.0 °C at 3000 RPM and 27.0 °C at 4800 RPM, meaning that the sludge temperature did not significantly affect the pretreatment process.

### 2.3 Sample preparation and analysis

Experiments were performed within 2 h of WAS and inoculum sampling. In total a sample of 60 L was poured into a larger container and mixed well to obtain homogeneous starting suspension for all experiments on the RGHS. For the RGHS 10 L of the well mixed WAS sample was used. Adequate amounts of samples were collected for analysis before and after the pretreatment experiments. All analyses were conducted in triplicate. For anaerobic digestion 10 L of inoculum was used to fill the reactors and for analyses. The efficiency and performance of the pretreatment regimes were compared by analysis of the physical and chemical properties of the WAS, rheological properties, scanning electron microscopy (SEM) analysis, Fourier transform infrared (FTIR) analysis and biomethane potential test.

#### 2.3.1 Analytical methods for evaluation of WAS samples

Physicochemical properties of WAS were measured before and after treatment on RGHS. Physical properties were measured by particle size and distribution analysis (analyses in triplicates), SEM analysis and rheological properties of WAS. The chemical properties were measured by total and/or soluble fraction analysis and FTIR analysis. More detailed descriptions are given in the following sections and in references [13,33].

#### 2.3.2 Dewaterability measurements

CST (Capillary Suction Time) is a common method used to evaluate the dewaterability of sludge. For the analysis of our WAS samples, we used a 304 M CST apparatus (Tritonel) together with 18 mm cylindrical cell. WAS sample was introduced into a cylindrical cell of the CST apparatus, which was equipped with filter paper placed on a smooth surface beneath the sample cell. The CST apparatus measured the capillary suction time by automatically recording the time required for the liquid to travel a set distance across the filter paper upon releasing the sludge sample. This measurement reflected the sludge’s resistance to filtration and indicated its dewaterability. Low CST values indicated that the sludge exhibited good dewaterability, as water was easily separated from the solid fraction, whereas high CST values suggested that the sludge had poor dewaterability [13].

#### 2.3.3 Analyses of released organic and inorganic compounds

To determine pH, redox potential, Electrical conductivity. Total dissolved solids (TDS) of WAS before and after treatment and inoculum, multimeter HQ4300 (Hach) was used. Soluble chemical oxygen demand (sCOD), total nitrogen (TN), and nitrogen species NH_4_–N were measured according to [33]. Total solids (TS), volatile solids (VS), total COD (tCOD), according to APHA Standard Methods [34].

#### 2.3.4 Infrared spectroscopy

To detect changes in chemical structure of processed WAS, attenuated total reflectance Fourier transform infrared (ATR-FTIR) was performed. Original and pretreated WAS samples were centrifuged at 15000 rpm for 10 min to obtain supernatant that was then further analyzed with ATR-FTIR Omega 2 (Perkin Elmer, USA) with wavenumber in range from 500 cm-1 to 4000 cm-1. Resolution was set to 1 cm^−1^. In total, 32 scans were performed, as described before [35].

#### 2.3.5 Scanning electron microscopy

The activated sludge samples were prepared for scanning electron microscopy (SEM) by centrifuging the liquid suspension obtained from the aerated pool. The resulting pellet was then resuspended in a 5 % glutaraldehyde solution prepared in 0.1 M phosphate buffer and processed. To examine the morphological properties of the activated sludge, the dried cells were mounted onto a SEM sample stub using double-sided carbon tape. The surface morphology was analyzed using a SUPRA 35VP scanning electron microscope (Carl Zeiss, Jena, Germany) under near-vacuum conditions, as described in [35].

#### 2.3.6 Size and distribution of particles

To measure particle size and distribution of untreated and treated WAS laser Particle Sizer Analyzer Analysette 22 – Wet dispersion unit (Fritsch, Germany) was used. First, the cumulative frequency distribution Q_3_ (x) and its derivative dQ_3_ (x) are displayed. The Q_3_ (x) curve provides information on the cumulative volume percentage of particles smaller than a specific size x, offering insight into the overall distribution of particle sizes within the sample. The derivative dQ_3_ (x) with respect to x represents the rate of change in the cumulative volume fraction as a function of particle size, highlighting the distribution's variation across different particle sizes. In addition to these, both the volume moment mean and specific percentiles (d_10_, d_50_, d_90_) are reported. The percentiles indicate the particle sizes below which 10 %, 50 % and 90 %, of the cumulative volume of particles are contained, respectively, providing key reference points within the particle size distribution. The volume moment mean, representing the average particle size weighted by volume, is calculated by summing the products of individual particle volumes and their respective sizes, then dividing by the total volume of the distribution [13,36].

#### 2.3.7 Rheological analysis

The Anton Paar MCR302 rheometer was used for rheological analysis of both untreated and pretreated WAS. The measurements were conducted using the CC39 measuring system and cup. The gap size, determined based on particle size distribution analysis, was set to 1 mm, and no additional screening of the samples was required as previously demonstrated in [3,13,33]. At the start of the test, the sample temperature was set to 25 °C with a tolerance of ± 0.1 °C. A waiting period of 60 s was selected for the sample to stay within this range before proceeding with the measurement. For the amplitude sweep test, shear strain (γ) was increased logarithmically from 0.01 % to 1000 % at a constant angular frequency (ω) of 1 rad/s. For the flow test, the shear rate ($\dot{\gamma }$) was linearly ramped from 1 to 1000 s^−1^ to obtain the flow curves.

#### 2.3.8 Methane potential test

Biochemical methane potential (BMP) was measured using the AMPTS II system (BPC Instruments, Sweden). The experiment utilized 0.5 L reactors placed in a thermostatic bath at 38 °C. A mixture of 350 ml of inoculum, sourced from the anaerobic reactor at WWTP Domžale-Kamnik, and 50 ml of substrate (WAS) was used. To capture CO_2_ and H_2_S, 80 ml of 3 M NaOH was placed in 100 ml flasks, which were connected in series between the 0.5L reactors and the measuring unit. In total 24 anaerobic reactors were used, all in triplicates as in our precious studies [3,6,7,13,33,36].

#### 2.3.9 Statistical analysis

A non-parametric multivariate analysis of variance (npANOVA and npMANOVA) was performed on the physicochemical parameters of both untreated and cavitated sludge samples using PAST software version 4.17c [37]. A significance level (p) of 0.05 was set to identify statistically significant differences. Following the npANOVA and npMANOVA, multidimensional scaling (nmMDS) analysis was conducted to visualize and interpret the data, revealing key patterns and relationships within the dataset as previously described in [6,7,13,36,38,39].

## 3 Results and discussions

### 3.1 Assessment of hydraulic shock and cavitation activity

In our experiments, the effects of hydraulic hammer-induced shock waves and hydrodynamic cavitation were only investigated post festum through observed changes in sludge properties. A direct assessment of the presence and intensity of these phenomena by methods such as high-speed imaging or hydrophone pressure measurements was not possible due to sludge opacity and technical challenges in mounting a pressure sensor close enough to the impact zone without substantially disrupting the jet or damaging the sensor. Furthermore, previous water hammer experiments [25,31] show that even if a transparent liquid is used to investigate impact-related phenomena, the jet quickly disintegrates into a curtain of fine droplets, thus hindering optical detection of shockwaves and cavitation within the liquid.

To address these issues, hydraulic hammer and HC phenomena were assessed using a numerical model by Wu et al. [29] for the impact of a cylindrical body of water against a flat surface, which closely resembles impacts occurring in the RGHS and is experimentally verified by a preceding study [26]. According to Wu et al. [29], the extent of HC is negligible for impact velocity below approximately 100 m/s. Since the largest impact velocity in the present study was 70 m/s, it is highly unlikely that cavitation, if at all present, played a significant role in the WAS pretreatment mechanism. Nevertheless, high-amplitude shock waves (few tens of MPa) can still be expected under such conditions [29].

To estimate the peak shockwave amplitude in the liquid domain *p*_max_, we can use the general hydraulic hammer equation *p* = *kρvc*, where *ρ* = 1000 kg/m^3^ is sludge density, *c* = 1500 m/s is the speed of sound in sludge (assumed to be the same to sonic speed in pure water), *v* is impact velocity and *k* is the ratio of *p*_MAX_ to theoretical water hammer pressure *ρvc* that has been estimated to *k* ≈ 0.27 from pressure distribution results presented in [29]. Using the peak shockwave pressure equation, we obtain: *p*_max_ = 28 MPa at *v* = 70 m/s and *p*_max_ = 18 MPa at v = 44 m/s. Such maximum pressure values can be expected along the cross-section centerline of a symmetric circular jet, while the distribution of pressure maxima in the jet’s cross section is nonuniform and tends to decrease away from the centerline [29] with mean maximum pressure exerted on the liquid of 1/3 to 1/2 of *p*_max_. Thus, it can be estimated that the sludge in this study was subjected to shockwaves with 6–14 MPa pressure amplitude in each pass, depending on the impact velocity. Considering that following the impact the liquid jet is dispersed to a spray of fine droplets, secondary impacts are possible when these droplets traverse the blade rotation zone. This can further enhance the sludge disintegration process, although at the expense of increased per-pass energy consumption.

### 3.2 Chemical changes in waste sludge samples

The pH of the sludge showed minimal variation across different pretreatment conditions, remaining within the range of 6.96 to 7.25 (Table 2). This indicates that the mechanical treatment process did not significantly alter the acidity or alkalinity of the waste-activated sludge (WAS). Oxidation-Reduction Potential (ORP) measures the tendency of a solution to either gain or lose electrons. Since it represents the activity of electrons, ORP is highly sensitive to changes in the redox balance, which is directly influenced by cellular metabolism, especially in microbial processes. This sensitivity makes ORP an important indicator of the metabolic state of microorganisms, as well as the surrounding biochemical environment [40]. Similarly to the pH, the ORP in this experiment decreased slightly with increasing passes (Np), from an initial value of 41 mV to 34 mV after 30 passes at 3000 RPM. This slight reduction in ORP suggests a gradual shift towards a more reducing environment, likely due to the breakdown of organic material, which consumes available oxidants that are formed during hydrodynamic cavitation [3,14,33].

**Table 2:** Chemical changes in pH, electrical conductivity (EC), redox potential, total dissolved solids (TDS), Salinity, Resistivity, soluble NH_4_-N, soluble PO_4_-P, and soluble COD before (0) and after treatments (R1-5 to R2-30).

**parameter**	**sample**						
	**0**	**R1-5**	**R1-15**	**R1-30**	**R2-5**	**R2-15**	**R2-30**
f (RPM)	/	3000	3000	3000	4800	4800	4800
Np	0	5	15	31	5	15	30
pH (−)	6.96 ± 0.08	7.06 ± 0	7.07 ± 0	7.08 ± 0.02	7.06 ± 0.01	7.17 ± 0.01	7.25 ± 0.01
EC (μS/cm)	1594 ± 26	1687 ± 11	1751 ± 13	1808 ± 29	1617 ± 16	1600 ± 8	1605 ± 8
redox (mV)	41 ± 5	36 ± 0	35 ± 0	34 ± 1	36 ± 0	29 ± 0	25 ± 1
TDS (mg/l)	693 ± 4	727 ± 3	763 ± 6	778 ± 3	696 ± 1	693 ± 3	698 ± 2
Salinity (‰)	0.69 ± 0.01	0.72 ± 0.01	0.78 ± 0.01	0.80 ± 0	0.70 ± 0.01	0.71 ± 0.01	0.70 ± 0
Resistivity (Ωcm)	711.67 ± 3.51	689.00 ± 4.00	648.33 ± 2.89	631.00 ± 1.73	709.67 ± 2.08	710.67 ± 1.15	714.00 ± 2.65
sNH_4_-N (mg/l)	83 ± 0	97 ± 2	105 ± 1	113 ± 1	91 ± 2	95 ± 1	93 ± 1
sPO_4_-P (mg/l)	11 ± 0	17 ± 1	23 ± 0	28 ± 1	13 ± 0	14 ± 0	17 ± 0
sCOD (mg/l)	1263 ± 81	2483 ± 11	2960 ± 21	3510 ± 21	1810 ± 42	2150 ± 0	2278 ± 18

The electrical conductivity (EC) of the sludge increased as the treatment progressed, with an increase of 13.4 % after 30 passes at 3000 RPM (Table 2). This rise in EC is indicative of the release of intracellular ions, such as potassium (K^+^), sodium (Na^+^), and chloride (Cl^−^), due to the mechanical disintegration of microbial cells. The pretreatment significantly increased the concentration of free ions in the sludge, enhancing its overall conductivity [41]. Conversely, the resistivity decreased as the number of passes increased, dropping from 711.67 Ωcm for untreated sludge to 631.00 Ωcm after 30 passes at 3000 RPM for R1, where impact velocity was 3000 RPM, reflecting an 11.4 % decrease. The reduction in resistivity corroborates the increase in EC for R1, further emphasizing the release of ions and the corresponding rise in sludge conductivity. On the other hand, in R2 chemical effects on EC and resistivity were negligible. It is likely that new binding sites were formed in the R2 regime to which the released ions bound. Consequently, the electrical conductivity and salinity did not change significantly.

Total dissolved solids (TDS) in R1 followed a similar increasing trend as EC, rising from 693 mg/L in untreated sludge to 778 mg/L (12.3 % increase) after 30 passes at 3000 RPM (Table 2). This increase suggests that pretreatment facilitates the solubilization of both organic and inorganic compounds, including salts and other cellular byproducts. Salinity also exhibited a slight but consistent increase, from 0.69 ‰ to 0.80 ‰, further indicating the release of solubilized ions during mechanical shearing [42]. However, no effects on mechanical pretreatment were noticed for R2.

Pretreatment led to a notable increase in nutrient concentrations, particularly ammonium and phosphate in R1. Ammonium (sNH_4_-N) concentrations rose 36.16 % after 30 passes at 3000 RPM. This rise can be attributed to the ammonification of nitrogenous organic compounds released from lysed cells. Phosphate (sPO_4_-P) concentrations also increased significantly, around 154.5 % after 30 passes. This release of inorganic phosphate likely stems from the disintegration of cellular phospholipids and nucleotides [43,44]. The increase in both ammonium and phosphate concentrations indicates that the mechanical disruption of sludge enhances the release of intracellular nutrients, which could have implications for subsequent biological or chemical treatment processes [44,45].

Soluble chemical oxygen demand (sCOD) increased substantially throughout the pretreatment process for 178 %, after 30 passes at 3000 RPM. The increase in sCOD reflects the solubilization of organic matter, primarily due to the mechanical disintegration of microbial cells. The sharp rise in sCOD indicates a high degree of sludge hydrolysis, with complex organic materials being broken down into simpler, soluble compounds [3,14,33,36]. This enhanced solubilization is critical for subsequent anaerobic digestion processes, as it improves the bioavailability of organic substrates for microbial degradation [3,5,14,36,46].

### 3.3 Physical changes in waste sludge samples

Hydraulic shock pretreatment significantly impacted the particle size distribution, specific surface area, and dewaterability of wastewater sludge, with distinct differences observed between the two operating regimes, R1 and R2 (Table 3).

**Table 3:** Percentiles, volume moment mean, specific surface area and capillary suction time before and after treatment.

**Parameter**	**Sample**						
	**0**	**R1-5**	**R1-15**	**R1-30**	**R2-5**	**R2-15**	**R2-30**
d10 (µm)	31.6	8.75	9.23	7.04	12.2	8.17	6.89
d50 (µm)	119	80.9	96.6	41.1	55.3	28.0	21.5
d90 (µm)	289	397	426	439	390	240	243
D[3,4] (µm)	169.4	156.3	168.1	218.3	144.8	101.53	59.1
Specific surface area (cm^2^/cm^3^)	1,381.411	3,237.390	3,036.376	3,440.785	2,611.657	4,007.561	5,367.514
CST (s)	62.3 ± 0.6	655.3 ± 50	782.5 ± 13	899.3 ± 40.1	410.1 ± 23.7	619.3 ± 28.4	746.4 ± 46.0

For the untreated sample, the d90 was 289 µm. Post-treatment, d90 increased to 397–439 µm for R1 and ranged between 240–390 µm for R2. This indicates that R1 underwent more pronounced re-aggregation of smaller particles into larger flocs compared to R2. The d50 decreased from 118 µm (untreated) to 41–80 µm for R1 and 21–55 µm for R2, demonstrating effective disruption of larger particles, especially in R2. The d10 value also decreased from 31 µm (untreated) to 7–9 µm for R1 and 6–12 µm for R2, reflecting a substantial reduction in the smallest particle sizes in both samples.

**Fig. 3:**
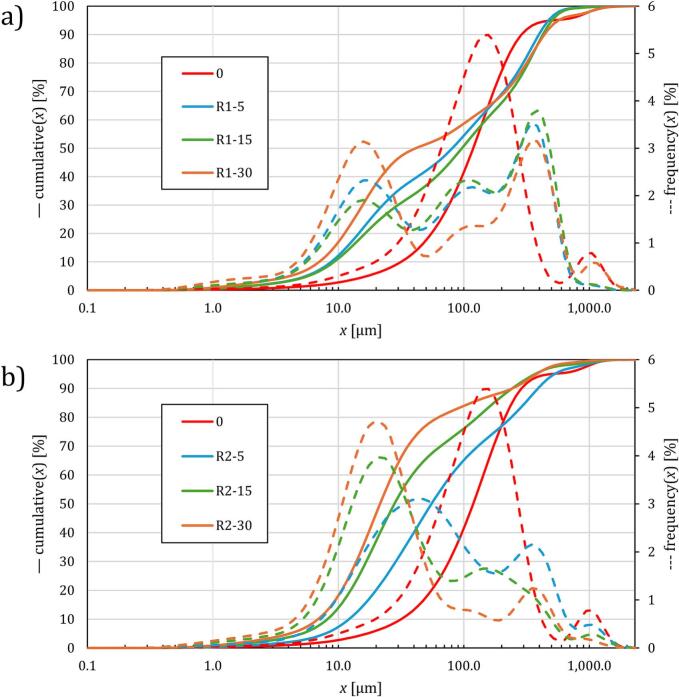
Particle size distribution for regimes R1 (a) and R2 (b).

This process led to an increase in the average particle size D[3,4], as smaller floc fragments coalesced into larger structures. Specifically, D[3,4] increased from 169 µm (untreated) to between 156–218 µm for R1 and decreased to 59–144 µm for R2. Fig. 3 illustrates the particle size distribution for pre-treated R1, showing two distinct peaks: one between 5 µm and 40 µm, and another between 200 µm and 700 µm, with a less distinct peak between 40 µm and 200 µm. This bimodal distribution suggests the presence of both fine fragments and re-aggregated larger flocs in the pretreated sludge as also reported before [36]. Statistical analysis revealed statistically significant differences (p < 0.0.5, F = 18.03) between average particle sizes between 0 and R2-30; R1-15 and R2-30; and R2-5 and R2-50.

The specific surface area of the untreated sludge was 1,381,411 cm^2^/cm^3^. After pretreatment, it increased significantly up to 3,440.785 cm^2^/cm^3^ for R1, reflecting an increase up to 208 %, and 2,611,657–5,367.514 cm^2^/cm^3^ for R2, representing an increase up to 388 %. This substantial increase in specific surface area indicates the generation of a higher number of smaller particles with a larger collective surface area, particularly pronounced in R2, where particle breakdown was more significant.

The Capillary Suction Time (CST) of the untreated sludge was 62.3 s. Following pretreatment, CST increased dramatically to between 768–947 s for both R1 and R2, reflecting an increase of 1139 % to 1427 %. This sharp increase indicates a marked reduction in dewaterability, likely due to the formation of more compact or gel-like structures that retain water more effectively, thereby complicating the dewatering process. Similar increase of CST was also found in our previous studies [3,33]. HS pretreatment, by breaking down floc structures into smaller particles, can increase the clogging of microfiltration media due to the higher fouling potential of these fine particles [47] and in that way increase the CST.

The observed changes can be attributed to biopolymers released during pretreatment. These biopolymers are believed to function as a “glue” that holds bioflocs together by forming functional groups such as hydroxyl and negatively charged carboxy groups. Hydraulic shock induced by the blade impacting the jet manifests as pressure waves propagating through sludge and disrupting existing floc structures. This disruption causes smaller particles to break apart, which then re-aggregate into larger flocs [36,48].

However, the CST increase in our case is not problematic since HS treatment was applied after thickening and polymer addition. This contrasts with the effects observed by Zhang et al. [49], where acid-AMD treatment improved dewaterability by altering EPS distribution.

HS pretreatment results in significant modifications to sludge properties. R1 shows more pronounced re-aggregation and higher average particle sizes, while R2 demonstrates greater particle breakdown. Both pretreatment regimes exhibit increased specific surface area and a notable decrease in dewaterability. The bimodal particle size distribution and the role of biopolymers in floc formation underscore the complex effects of HS on sludge structure and processing. These findings highlight the need for potential adjustments in sludge management strategies to address the challenges introduced by HS pretreatment.

**Fig. 4:**
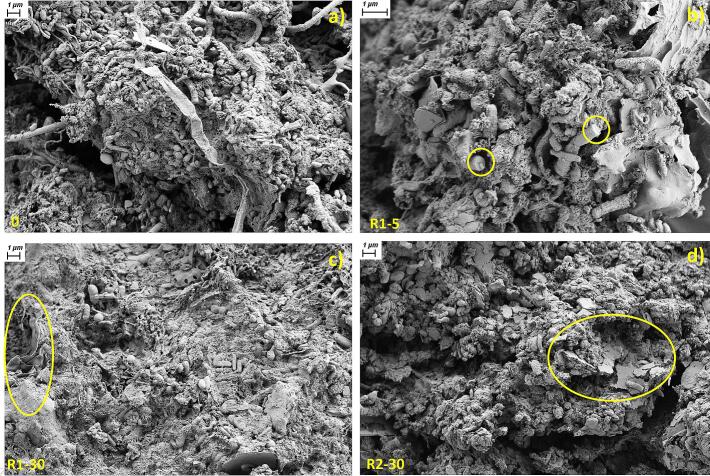
SEM images showing sludge flock morphology after HS treatment: (a) untreated WAS; (b) R1-5; (c) R1-30; (d) R2-30.

Further changes in the shape of the WAS can also be seen in the SEM images. Fig. 4 shows the changes in the shape and surface area of the sludge under different pre-treatment options. In Fig. 4a, which shows the untreated sludge, we can clearly see the round, rod, spiral and filamentous micro-organisms forming flocs. The pre-treatment of the sludge has resulted in damage and detachment of the filamentous micro-organisms (Fig. 4b). As the Np number increases, we can see that there are fewer micro-organisms on the surface of the floc, and damage to the exterior or surface of the floc is also visible (Fig. 4c). Surface damage and deformation similar to blunt force trauma is clearly visible for R2-30 (Fig. 4d). All this further confirms the mechanical effects of impact-induced HS on WAS.

### 3.4 Rheometer measurements

**Fig. 5:**
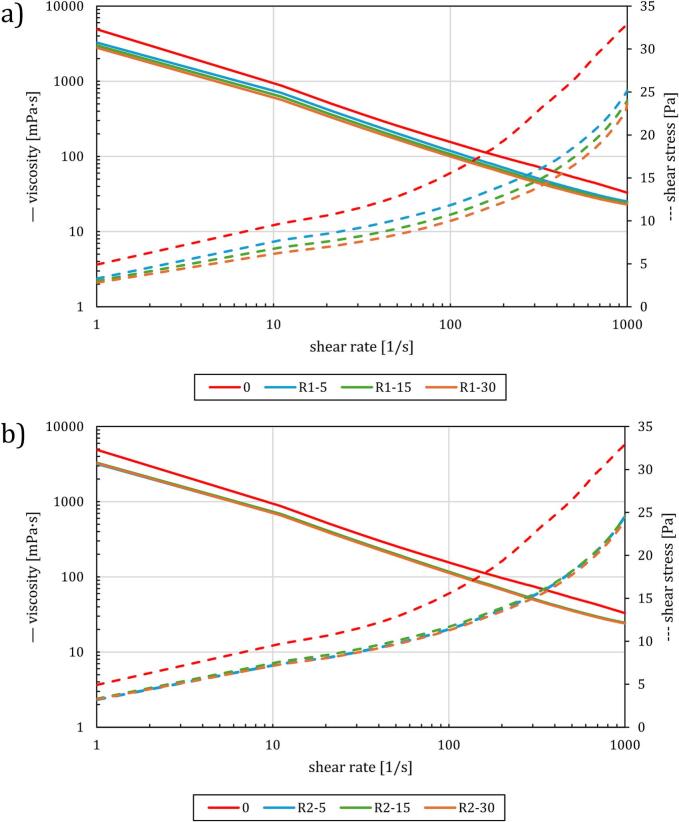
Apparent viscosity as a function of shear rate for 4800 RPM conditions (above) and 3000 RPM conditions (below).

Flow test results in Fig. 5 indicate a shear-thinning behavior of processed and unprocessed sludge samples. Under both operating conditions, sludge treatment reduces apparent (shear-dependent) viscosity across the complete measurement range. Viscosity reduction of about 60 % (e.g., from 5 Pa∙s to 2 Pa s at 1/s shear rate) is observed when initial WAS is subjected to 30 cycles of RGHS treatment.

**Fig. 6:**
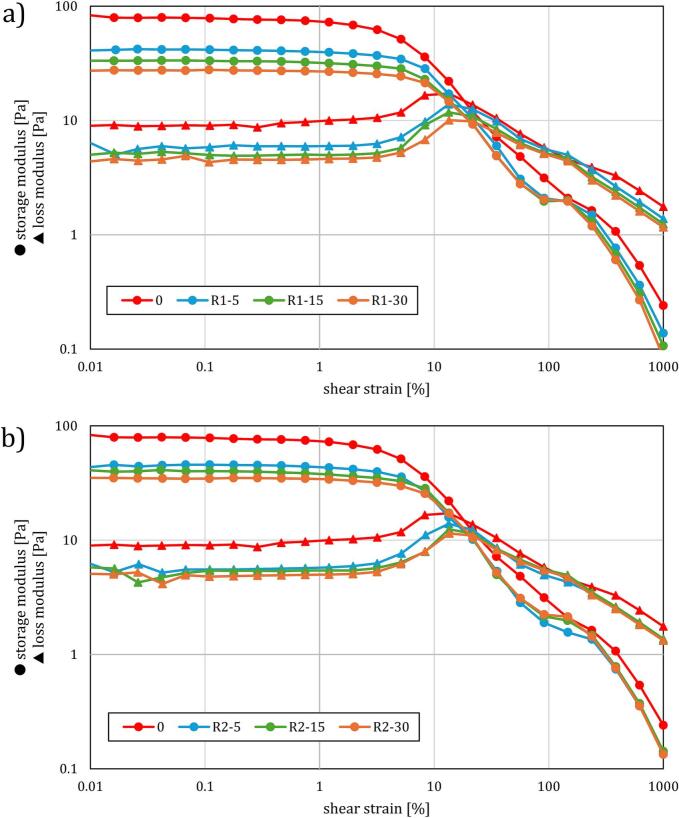
Amplitude sweep test diagrams for regimes R1 (a) and R2 (b).

The amplitude sweep test results depicted in Fig. 6 reveal key insights into the viscoelastic behavior of untreated and treated WAS samples under varying shear strains. The untreated sludge consistently exhibits higher storage modulus (G') and loss modulus (G“), indicating a robust elastic network with strong internal bonding that helps maintain its form under stress. After undergoing HS treatment, both moduli decrease significantly, particularly in the lower shear strain range (0.01 % to 10 %). This suggests that the treatment disrupts the sludge's internal network, reducing its structural integrity and leading to a more fluid-like behavior. At higher shear strains, the treated samples show a convergence of G' and G”, indicating a transition from elastic to more viscous behavior, with the most pronounced effects observed with the samples subjected to the highest number of passes (R1-30 and R2-30).

In regime 2, where HS was less intense due to slower rotor blade movement (3000 RPM), the reductions in G' and G“ are less pronounced compared to regime 1. The treated samples in regime 2 retain more of their original viscoelastic properties, especially at lower shear strains (0.01 % to 10 %), indicating that the weaker treatment causes less disruption to the sludge's structure. When comparing both regimes, it is evident that the intensity of HS plays a crucial role in modifying the sludge's rheological behavior. Regime 1, with its higher energy input, leads to more substantial structural changes, making the sludge more susceptible to deformation and flow, while regime 2 resulted in more moderate changes, preserving more of the sludge's original structure.

The effects of HS treatment on G' and G'' align with findings by Zhang et al. [50], who observed that acidification and anaerobic mesophilic digestion (acid-AMD) reduced G' and G'', thereby weakening the sludge's internal structure. This study highlighted the effective disruption of extracellular polymeric substances (EPS), which are crucial for sludge dewaterability. While both HS and acid-AMD treatments resulted in a reduction of G' and G'', indicating a transition to more viscous behavior, our CST results show that HS treatment worsened dewaterability. This suggests that HS treatment might also disrupt EPS similarly to acid-AMD, but the release of excess biopolymer into solution could further deteriorate dewatering performance [51]. Thus, while both treatments reduce G' and G'', HS treatment appears to exacerbate dewatering issues by potentially releasing more biopolymer.

### 3.5 FTIR spectra analysis

**Fig. 7:**
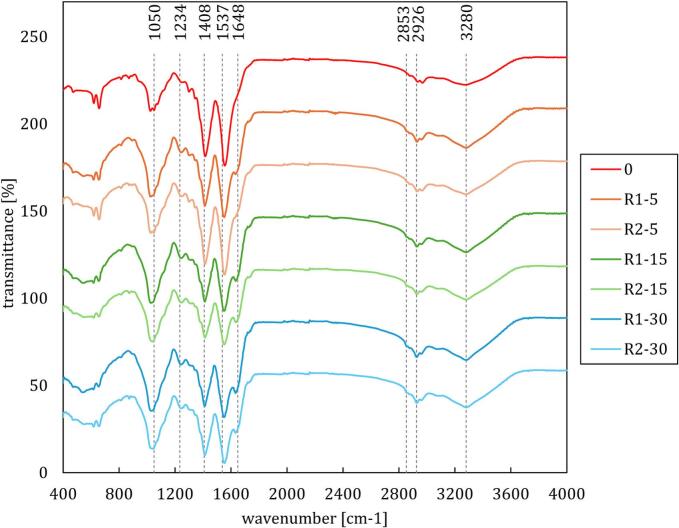
FTIR spectra of analyzed WAS samples.

In the following, the FTIR spectra of the supernatant samples are presented (Fig. 7). The region around 3200–3600 cm^−1^ corresponds to O-H stretching vibrations, associated with water content and hydrogen-bonded hydroxyl groups in organic matter. A slight decrease in intensity and alteration in shape after treatment suggests potential dehydration or disruption of hydrogen bonds within the cellular structure.

The peaks in the 2800–3000 cm^−1^ range, representing C-H stretching vibrations of aliphatic hydrocarbons (such as lipids and fatty acids), showed minimal changes. This indicates that the aliphatic content remained relatively stable, though subtle alterations hinted at minor lipid degradation or transformation.

In the 1500–1700 cm^−1^ region, which is characteristic of Amide I (C=O stretching) and Amide II (N-H bending) vibrations linked to protein content, a noticeable reduction in peak intensity was observed in treated samples. This reduction signified protein denaturation or breakdown due to hydraulic shock treatment, which is a strong indicator of cell wall damage [13].

The 1000–1300 cm^−1^ region, associated with C-O stretching in polysaccharides and C-O-C linkages in carbohydrates, exhibited changes that particularly included reduced peak intensities in treated samples. This suggests degradation of polysaccharides or breakdown of extracellular polymeric substances (EPS) [13].

The regions around 1230 cm^−1^ (C-O stretching of carboxyl groups) and 1400–1450 cm^−1^ (CH2 bending) are crucial for assessing cell wall damage. These regions are also linked to amino acids that form microbial cell walls, with significant peaks at 1648 cm^−1^ and 1537 cm^−1^ [52], as well as a characteristic band at 1234 cm^−1^ [53], indicative of bacterial cell walls in both Gram-positive and Gram-negative bacteria. The decreased intensity and shifts in these peaks, especially in R1-30p, indicated substantial cell wall damage, likely resulting from the shear forces and pressure differentials experienced during treatment.

The FTIR analysis indicated that hydraulic shock treatment induced significant structural changes in WAS, with more pronounced effects observed in the R1 regime. Both R1 and R2 lead to protein denaturation, polysaccharide degradation, and disruption of cellular structures, which are key indicators of effective cell lysis. However, the R1 regime showed more substantial changes, including greater breakdown of proteins and polysaccharides (1000–1300 cm^−1^) and more significant cell wall damage (1230 cm^−1^ and 1400–1450 cm^−1^). These changes suggest that more aggressive treatment in R1, likely due to higher energy input, was more effective in altering the composition and structure of WAS, with potential benefits for improving downstream processes such as enhanced biodegradability.

### 3.6 Methane production

**Fig. 8:**
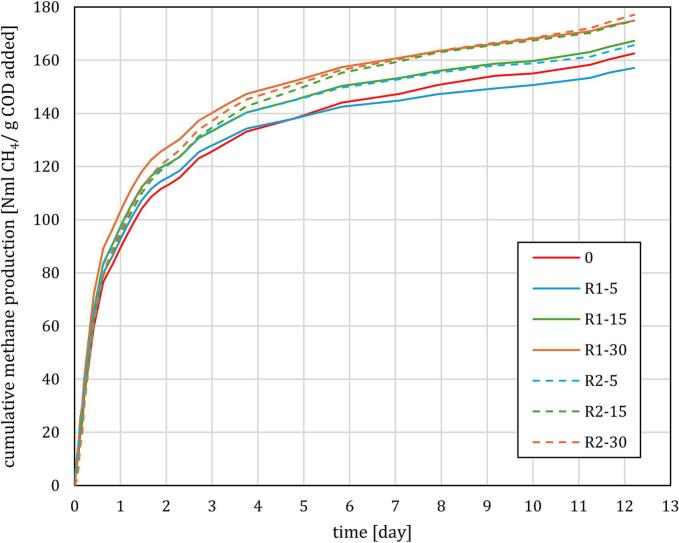
Cumulative methane production for initial sample 0 and all 6 treated samples.

The biomethane potential test was run for 12 days, with cumulative methane production (CMP) per mass of COD added shown in Fig. 8. Compared to the initial sample (sample 0), higher methane production was measured in all pretreated samples except for R1-5 where methane production underperformed the untreated sample after day 5. Also, around this time, the daily production rate of all the other samples stabilized at about 3 NmL CH_4_/g COD added/day until the end of measurements.

As seen from Fig. 8, CMP enhancement relative to baseline (sample 0) increased with the number of sludge-passes through the RGHS. The highest CMP (namely, 177.1 ± 4.4 NmL CH_4_/g COD added) was observed for regime R2 at Np = 30, representing 9.0 % increase relative to sample 0 (CMP = 162.5 ± 8.7 NmL CH_4_/g COD added). Sample R2 at 15 passes and R1 at 30 passes produced only slightly lower CMP of 174.9 ± 4.8 NmL CH_4_/g COD added, or 7.6 % improvement over sample 0. Therefore, to consistently improve CMP, WAS should be subjected to at least 15 passes of pretreatment. For comparison, using a pinned-disc RGHC, Zupanc et al. [3] measured approximately 20 % relative increase in methane formation after 60 passes over unprocessed sample, meaning approximately the same per-pass effect (i.e., about 10 % improvement after 30 passes). Economic analysis of methane production comparative to other types of WAS mechanical pretreatment devices is presented in Table 4.

**Table 4:** Comparison of WW pretreatment effectiveness and energy efficiency for different processing setups (best case values provided for all comparative studies). All studies performed treatment of WAS samples, with present study utilizing dewatered WAS and Vilarroig et al. [18] a mixture of WAS and pig slurry.

**study**	**setup**	**V (L)**	**P (kW)**	**pH**	**TS init. (g/L)**	**TCOD (g/L)**	**ΔsCOD (g/L)**	**t (min)**	**Np**	**DD (%)**	**SEC, KWh/ kg sCOD**	**SMY L/kWh**
present	RGHS R1	10	1.15	7.0	36	27.5	1.22	2.8	5	22.4	4.36	−4.70
present	RGHS R1	9*	1.15	7.1	36	27.5	1.70	7.22	15	31.2	8.16	4.07
present	RGHS R1	8*	1.15	7.1	36	27.5	2.25	12.2	30	41.3	10.4	10.6
present	RGHS R2	10	0.40	7.1	36	27.5	0.547	2.8	5	10.1	3.39	7.74
present	RGHS R2	9*	0.40	7.2	36	27.5	0.887	7.22	15	16.3	5.43	30.2
present	RGHS R2	8*	0.40	7.3	36	27.5	1.02	12.2	30	18.7	8.03	33.1
[13]	PD RGHC	4	0.49	5.1	47	64.5	0.96	5.3	30	9.6	11.3	∼0
[3]	PD RGHC	5	0.57	6.8	17.9	9.4	0.475	12	60	22.3	47.8	15.8
[18]	SD RGHC	500	6.93	7.2	70	51.7	0.962	240	48	17.4	57.6	N/A
[55]	swirling jet HC reactor	50	3.0	6.5	43.5	24.0	0.617	60	40	1.4	97.2	N/A
[54]	orifice plate HC	25	1.5	7.5	14	13.2	6.76	150	78	60	22.2	1.86

*average volume during experiment, volume reduced from 10 L to 6 L by sludge sampling at 5 and 15 passes.

PD = pinned disc, SD = serrated disc.

### 3.7 Statistical analysis

Pearson‘s linear correlation analysis (Table S1) revealed that there are statistically significant correlations (p < 0.05, R > 0.79) between soluble chemical parameters (EC, ORP, TDS, salinity, resistivity, sCOD, sTOC, sNH_4_-N, sPO_4_) of WAS, what corresponds very well with the results of experiment. Further, statistical analysis using npANOVA and Kruskal-Wallis test revealed that there was significant difference between sample medians (p < 0.0.5, chi^2^ = 529.1). Therefore, we also performed Mann Whitney pairwise post-hoc test with sequential Bonferroni p values to compare groups. There was a significant difference (p < 0.0.5) for majority of physical–chemical parameters (Supplementary Table S2).

Similarly to the ANOVA, PERMANOVA calculates a pseudo-F statistic to compare the variation between groups to the variation within groups. In PAST [37] we used one-way PERMANOVA (npMANOVA) to compare the differences between pretreated samples (Table S3). Bonferroni corrected p values showed that there is a statistically significant difference (p < 0.0.5, F = 169.2) between R1 and R2 physical–chemical parameters. The difference was also statistically significant within R2 operating conditions (R2-5 to R2-30). Significant differences indicated distinct effects of their respective pretreatment operations. The significant difference between these pretreatment operation regime groups indicates that the pretreatment methods applied to these groups resulted in distinct physical–chemical characteristics. This could mean that the specific pretreatment processes have a substantial impact on the R2 physical–chemical parameters. There are no statistically significant differences within R1-5 to R1-30 (Table S2, Table S3). The pretreatment processes were effective in altering the physical–chemical parameters compared to the untreated samples (p < 0.05, F = 460.9).

**Fig. 9:**
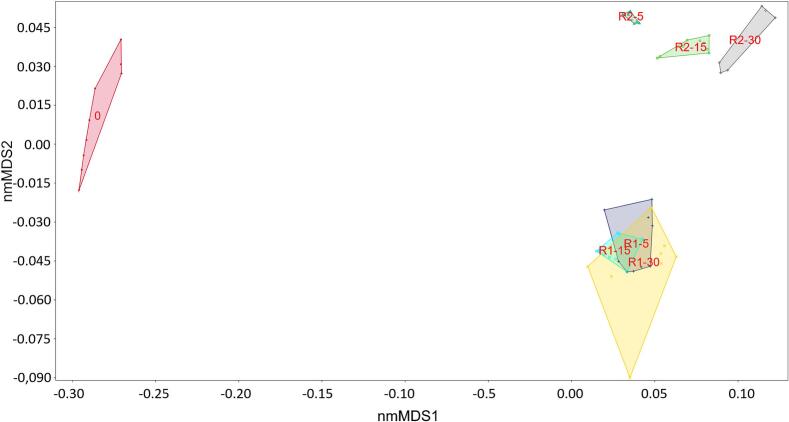
Non-metric multidimensional scaling analysis for physio-chemical parameters for the untreated (0) and pretreated samples.

A nm-MDS analysis was performed to further assess the physicochemical parameters of wastewater sludge and reveals a significant overlap of the measured physicochemical parameters between different treatments. The emergence of overlying and overlapping convex shells led to the identification of three distinct groups (Fig. 9). The first group was the untreated sludge, the second group included the pretreated sludge (R1-5 to R1-30), and the third group included the R2-5 to R2-30 pretreated sludge. The stress value for this analysis was calculated as 0.1585, with R2 values of 0.8642 for the x-axis and 0.03337 for the y-axis. Overlap of R1-5 to R1-30 confirmed that physicochemical parameters of these pretreatment regimes are more similar to each other.

### 3.8 Pretreatment effectiveness and energy efficiency

To assess the performance and energy efficiency of the RGHS relative to devices used in other studies, comparison of key operating characteristics is provided in Table 4. As evident, the present study setup exhibited significantly lower specific energy consumption (SEC, Eq. (1)) of sludge solubilization (3.4–10.4 KWh/kg sCOD released) compared to other studies where corresponding energy consumption was between 11.3 and 97.2 KWh/kg sCOD released (PD RGHC performed best while the swirling jet HC reactor consumed most energy per mass unit of released sCOD). SEC in 30 passes was comparable to the PD RGHC experiment by [13], but the present RGHS setup was able to achieve a significantly higher disintegration degree (DD, Eq. (2)), namely 330 % higher at R1 conditions and 95 % higher at R2 conditions.

(1) $$\mathrm{SEC}=\frac{E}{\Delta sCOD}=\frac{P∙t}{{\mathrm{sCOD}}_{1}-{\mathrm{sCOD}}_{0}}$$

(2) $$\mathrm{DD}=\frac{\Delta sCOD}{{\Delta sCOD}_{\mathrm{NaOH}}}=\frac{{\mathrm{sCOD}}_{1}-{\mathrm{sCOD}}_{0}}{{\Delta sCOD}_{\mathrm{NaOH}}}$$

In Eqs. (1), (2), sCOD_0_ and sCOD_1_ refer to initial and final (Np passes-treated) sCOD of WAS as measured in our experiments. In Eq. (2), ΔsCOD_NaOH_ denotes the maximum possible increase of sCOD that was measured by addition of NaOH to unprocessed WAS. The invested processing energy *E* is equal to the product of electric power *P* drawn by RGHS, and WAS processing time *t*.

Interestingly, SEC at 5 and 30 liquid passes only increased by approximately 30 % when rotational speed of the was raised from 3000 RPM to 4800 RPM despite the increase in power consumption by 2.88 times (188 %). Considering the much larger DD at same Np, R1 regime (4800 RPM) are more suitable for practical application, especially since a roughly linear growing trend of DD in time was observed, indicating the possibility of exceeding 50 % disintegration degree upon continued treatment. On the other hand, the DD growth of the R2 regime slowed considerably after 15 liquid passes, only increasing from 16.3 % to 18.7 % in the last 15 passes. Compared to other studies in Table 4, the present study setup demonstrates comparatively higher disintegration degree in fewer liquid passes, achieving DD = 22.4 % in just 5 passes and DD = 41.3 % in 30 passes under R1 regime. Only the orifice plate experiment by Şaǧban et al. [54] was able to attain a higher DD (namely, 60 %), albeit at a much higher SEC and processing time.

To evaluate the economic efficiency of methane production enhancement, specific methane yield (SMY) was introduced by Eq. (3) as required energy investment *E* per increase of methane volume (namely, difference between V_CH4,1_ of processed WAS, and V_CH4,0_ of unprocessed WAS).

(3) $$\mathrm{SMY}=\frac{{\Delta V}_{CH4}}{E}=\frac{V_{\mathrm{CH}4,1}-V_{\mathrm{CH}4,0}}{P∙t}$$

Our RGHS device of 10.6 L CH4 per kWh for R1 conditions and 33.1 L/kWh for R2 conditions, at Np = 30. SMY at 5 passes was minimal for R2 and even slightly negative for R1 but increased significantly at 15 and 30 passes. For comparison, PD RGHC in a study by Zupanc et al. [3] produced SMY of 15.8 kWh/L at Np = 30, while a very similar setup by [13] only saw negligible SMY, likely due to acidic pH.

If methane production enhancement is the primary goal of RGHS operation, then regime R2 (i.e., slower blade impact velocity) with Np ≥ 15 is preferrable to R1, yielding a similar extra volume of methane at significantly lower energy input, thus resulting in much higher SMY. This implies that that a more aggressive processing regime that better disintegrates the sludge does not necessarily yield more biogas, nor is it necessarily more energy efficient than a more moderate one – in our case, R2 with a lower blade speed. Further investigation is required so that the RGHS operating parameters can be optimized.

The sludge processing capacity of presented RGHS system depends on the objectives of WAS treatment – sludge disintegration and solubilization on one hand, or methane production enhancement on the other. Operating regime 2 for 15 passes (Table 4) where low SEC is combined with high SMY and moderate DD, an effective treatment capacity was approximately 70 L/h of WAS. Although the sludge throughput in commercial WWTPs is typically significantly higher than this figure, RGHS devices can be easily scaled-up from laboratory to pilot or industrial scale implementing one or more of the following measures [31]: a) increasing the diameter (and hence, flow rate) of the water jet; b) increasing the thickness of the blade (i.e., larger volume of liquid processed in each impact); c) increasing the number of liquid jets (i.e. arranging several jets in a circular pattern about the blade axis).

It is worth noting that SMY > 90.4 L/kWh is required (calculated from higher heating value of methane) if we want the energy content of additionally released methane to exceed the consumed energy for operating the RGHS. Therefore, none of the WAS pretreatment setups compared in Table 4 can operate profitably if its sole function is the enhancement of methane production. Nevertheless, profitability can be achieved if the benefit of additionally produced methane is combined with improved anaerobic digestion, in turn reducing the mass of residual WAS requiring costly disposal by third parties.

## 4 Conclusions

In this paper, the effectiveness of a novel WAS pretreatment method by recurring hydraulic shocks was investigated. As indicated by the sludge properties assay, the novel RGRS device could achieve over 40 % sludge disintegration degree while consuming approximately 10 kWh energy per kg of released sCOD. Methane production was enhanced by up to 9 % in 30 liquid passes, yielding 33.1 L of additional gas per kWh of energy invested in the optimal case. Despite the absence of cavitation, hydraulic hammer-induced pressure shock waves proved an effective means of disintegrating WAS, inducing statistically significance changes in physicochemical parameters as demonstrated by high amounts of released sCOD, as well as changes in sludge rheology, particle size and FTIR spectra and SEM. These characteristics are remarkable in comparison with HC-based devices, and only PD RGHC devices have so far demonstrated a similar energy efficiency of sludge solubilization and biomethane production enhancement. Given the design of device and methodology of inducing hydraulic hammer in processed sludge, good scalability can be expected since additional sludge jets can be incorporated to the reactor vessel, effectively increasing the treatment capacity. Future research of the RGHS concept are warranted to include optimization of the blade impact surface shape to amplify the shock wave magnitude at a given impact velocity, possibly reducing the energy consumption of the device. Higher speed impacts generating HC are also withing the scope of investigation, with an understanding that the power demand of the device would drastically increase and would only be economically justified if WAS degradation can be achieved in much fewer liquid passes than presently.

## CRediT authorship contribution statement

**Sabina Kolbl Repinc:** Supervision, Methodology, Investigation, Funding acquisition, Writing – original draft. **Gašper Rak:** Writing – review & editing, Validation, Data curation. **Blaž Stres:** Validation, Resources. **Uroš Novak:** Methodology, Formal analysis. **Blaž Likozar:** Validation, Resources. **Anže Prašnikar:** Methodology, Data curation. **Marko Blagojevič:** Writing – original draft, Resources, Investigation, Data curation. **Benjamin Bizjan:** Writing – original draft, Project administration, Investigation, Conceptualization.

## Declaration of competing interest

The authors declare that they have no known competing financial interests or personal relationships that could have appeared to influence the work reported in this paper.
