# Supplementary material for: Pretreatment of waste activated sludge by rotational generator of hydraulic shock
Source: Ultrason Sonochem. 2025 Mar 13;116:107312. doi: 10.1016/j.ultsonch.2025.107312 (PMC11957678; doi:10.1016/j.ultsonch.2025.107312)
Supplement: Supplementary Data 1 [file mmc1.docx]

**Electronic Supplementary material**

**Pretreatment of waste activated sludge by rotational generator of hydraulic shock**

Sabina Kolbl Repinc^1,2^, Gašper Rak^1^, Blaž Stres^1,2,3^, Uroš Novak^2^, Blaž Likozar^2^, Anže Prašnikar^2^, Marko Blagojevič^1^, Benjamin Bizjan^*1,4^

^1^ University of Ljubljana, Faculty of Civil and Geodetic Engineering, Jamova 2, 1000 Ljubljana, Slovenia

^2^National Institute of Chemistry, Hajdrihova 19, 1000 Ljubljana, Slovenia

^3^University of Ljubljana, Biotechnical Faculty, Jamnikarjeva 101, 1000 Ljubljana

^4^ University of Ljubljana, Faculty of Mechanical Engineering, Aškerčeva cesta 6, 1000 Ljubljana, Slovenia

*Corresponding author. Email address: benjamin.bizjan@fs.uni.lj.si

Table S1: Pearson correlation matrix for physico-chemical parameters where p values<0.05 are boxed.

 
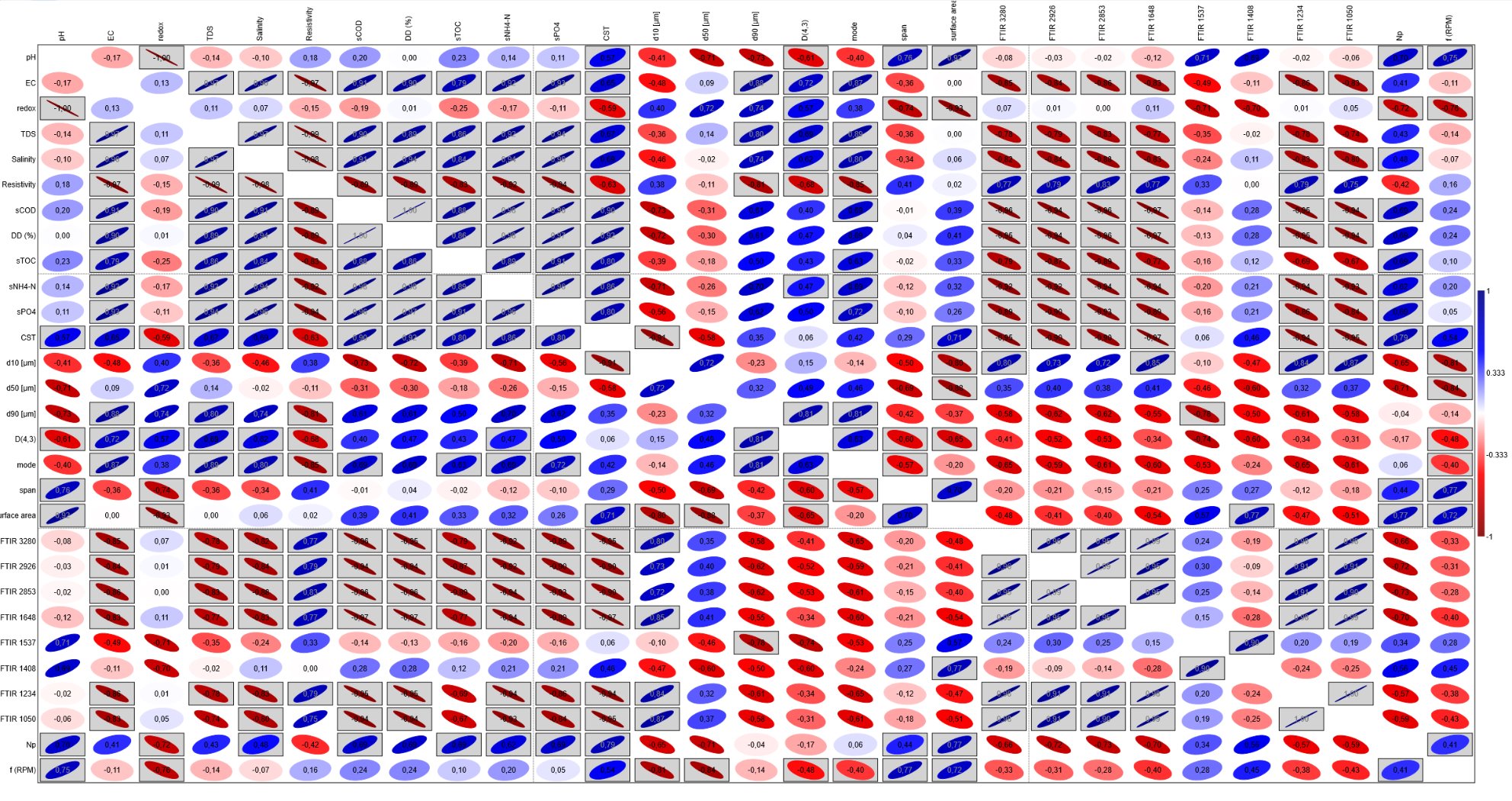


Table S2: Mann Whitney pairwise test for physico-chemical parameters with sequential Bonferroni p values.

|  | **pH** | **EC** | **redox** | **TDS** | **Salinity** | **Resistivity** | **sCOD** | **DD (%)** | **sTOC** | **sNH4-N** | **sPO4** | **CST** | **d10 [µm]** | **d50 [µm]** | **d90 [µm]** | **D(4,3)** | **mode** | **span** | **surface area** | **FTIR 3280** | **FTIR 2926** | **FTIR 2853** | **FTIR 1648** | **FTIR 1537** | **FTIR 1408** | **FTIR 1234** | **FTIR 1050** | **Np** | **f (RPM)** |
| --- | --- | --- | --- | --- | --- | --- | --- | --- | --- | --- | --- | --- | --- | --- | --- | --- | --- | --- | --- | --- | --- | --- | --- | --- | --- | --- | --- | --- | --- |
| **pH** |  | **0,00000** | **0,00000** | **0,00000** | **0,00000** | **0,00000** | **0,00000** | **0,00552** | **0,00000** | **0,00000** | **0,00000** | **0,00000** | 0,07824 | **0,00010** | **0,00010** | **0,00000** | **0,00000** | **0,00128** | **0,00000** | **0,00010** | **0,00010** | **0,00010** | **0,00010** | **0,00010** | **0,00010** | **0,00010** | **0,00010** | 0,49400 | **0,00000** |
| **EC** | **0,00000** |  | **0,00000** | **0,00000** | **0,00000** | **0,00000** | **0,00056** | **0,00011** | **0,00000** | **0,00000** | **0,00000** | **0,00000** | **0,00011** | **0,00011** | **0,00011** | **0,00000** | **0,00000** | **0,00000** | **0,00001** | **0,00011** | **0,00011** | **0,00011** | **0,00011** | **0,00011** | **0,00011** | **0,00011** | **0,00011** | **0,00000** | **0,00000** |
| **redox** | **0,00000** | **0,00000** |  | **0,00000** | **0,00000** | **0,00000** | **0,00000** | **0,01080** | **0,00000** | **0,00000** | **0,00000** | **0,00000** | **0,00037** | 0,08924 | **0,00011** | **0,00000** | **0,00132** | **0,00000** | **0,00000** | **0,00011** | **0,00011** | **0,00011** | **0,00011** | **0,00025** | **0,00027** | **0,00011** | **0,00011** | **0,00000** | **0,00000** |
| **TDS** | **0,00000** | **0,00000** | **0,00000** |  | **0,00000** | 0,06970 | **0,00000** | **0,00010** | **0,00273** | **0,00000** | **0,00000** | 0,19040 | **0,00010** | **0,00010** | **0,00010** | **0,00000** | **0,00000** | **0,00000** | **0,00000** | **0,00010** | **0,00010** | **0,00010** | **0,00010** | **0,00010** | **0,00010** | **0,00010** | **0,00010** | **0,00000** | **0,00000** |
| **Salinity** | **0,00000** | **0,00000** | **0,00000** | **0,00000** |  | **0,00000** | **0,00000** | **0,00532** | **0,00000** | **0,00000** | **0,00000** | **0,00000** | **0,00009** | **0,00009** | **0,00009** | **0,00000** | **0,00000** | **0,00000** | **0,00000** | **0,00009** | **0,00009** | **0,00009** | **0,00009** | **0,00009** | **0,00009** | **0,00009** | **0,00009** | **0,00000** | **0,00000** |
| **Resistivity** | **0,00000** | **0,00000** | **0,00000** | 0,06970 | **0,00000** |  | **0,00000** | **0,00010** | **0,00049** | **0,00000** | **0,00000** | 0,54580 | **0,00010** | **0,00010** | **0,00010** | **0,00000** | **0,00000** | **0,00000** | **0,00000** | **0,00010** | **0,00010** | **0,00010** | **0,00010** | **0,00010** | **0,00010** | **0,00010** | **0,00010** | **0,00000** | **0,00000** |
| **sCOD** | **0,00000** | **0,00056** | **0,00000** | **0,00000** | **0,00000** | **0,00000** |  | **0,00030** | **0,00000** | **0,00000** | **0,00001** | **0,00000** | **0,00030** | **0,00030** | **0,00030** | **0,00000** | **0,00000** | **0,00000** | **0,00471** | **0,00030** | **0,00030** | **0,00030** | **0,00030** | **0,00030** | **0,00030** | **0,00030** | **0,00030** | **0,00000** | **0,00055** |
| **DD (%)** | **0,00552** | **0,00011** | **0,01080** | **0,00010** | **0,00532** | **0,00010** | **0,00030** |  | **0,00011** | **0,00010** | 0,68160 | **0,00011** | 0,15990 | **0,01519** | **0,00217** | **0,00003** | **0,00083** | **0,00350** | **0,00002** | **0,00217** | **0,00217** | **0,00217** | **0,00217** | **0,00217** | **0,00217** | **0,00217** | **0,00217** | 0,10940 | **0,00100** |
| **sTOC** | **0,00000** | **0,00000** | **0,00000** | **0,00273** | **0,00000** | **0,00049** | **0,00000** | **0,00011** |  | **0,00000** | **0,00000** | **0,00098** | **0,00011** | **0,00011** | **0,00011** | **0,00000** | **0,00000** | **0,00000** | **0,00000** | **0,00011** | **0,00011** | **0,00011** | **0,00011** | **0,00011** | **0,00011** | **0,00011** | **0,00011** | **0,00000** | **0,00000** |
| **sNH4-N** | **0,00000** | **0,00000** | **0,00000** | **0,00000** | **0,00000** | **0,00000** | **0,00000** | **0,00010** | **0,00000** |  | **0,00000** | **0,00008** | **0,00010** | **0,03821** | **0,00010** | **0,00001** | 0,33890 | **0,00000** | **0,00000** | **0,00016** | **0,00030** | **0,00485** | **0,00010** | **0,00010** | **0,00010** | **0,00020** | **0,00010** | **0,00000** | **0,00000** |
| **sPO4** | **0,00000** | **0,00000** | **0,00000** | **0,00000** | **0,00000** | **0,00000** | **0,00001** | 0,68160 | **0,00000** | **0,00000** |  | **0,00000** | **0,01532** | **0,00117** | **0,00030** | **0,00000** | **0,00000** | **0,00000** | **0,00000** | **0,00030** | **0,00030** | **0,00030** | **0,00030** | **0,00030** | **0,00030** | **0,00030** | **0,00030** | 0,15310 | **0,00002** |
| **CST** | **0,00000** | **0,00000** | **0,00000** | 0,19040 | **0,00000** | 0,54580 | **0,00000** | **0,00011** | **0,00098** | **0,00008** | **0,00000** |  | **0,00011** | **0,00068** | **0,01696** | **0,00000** | **0,00000** | **0,00000** | **0,00000** | **0,00580** | **0,00580** | **0,00580** | **0,00297** | **0,00011** | **0,00011** | **0,00580** | **0,00020** | **0,00000** | **0,00000** |
| **d10 [µm]** | 0,07824 | **0,00011** | **0,00037** | **0,00010** | **0,00009** | **0,00010** | **0,00030** | 0,15990 | **0,00011** | **0,00010** | **0,01532** | **0,00011** |  | **0,00494** | **0,00217** | **0,00002** | **0,00007** | **0,00264** | **0,00002** | **0,00217** | **0,00217** | **0,00217** | **0,00217** | **0,00217** | **0,00217** | **0,00217** | **0,00217** | 0,80870 | **0,00142** |
| **d50 [µm]** | **0,00010** | **0,00011** | 0,08924 | **0,00010** | **0,00009** | **0,00010** | **0,00030** | **0,01519** | **0,00011** | **0,03821** | **0,00117** | **0,00068** | **0,00494** |  | **0,00217** | **0,00062** | 0,18480 | **0,00002** | **0,00002** | 0,60930 | 0,44330 | 0,20130 | 0,70150 | 0,79830 | 0,70150 | 0,60930 | 1,00000 | **0,00016** | **0,00142** |
| **d90 [µm]** | **0,00010** | **0,00011** | **0,00011** | **0,00010** | **0,00009** | **0,00010** | **0,00030** | **0,00217** | **0,00011** | **0,00010** | **0,00030** | **0,01696** | **0,00217** | **0,00217** |  | **0,00003** | **0,00595** | **0,00002** | **0,00002** | **0,00217** | **0,00217** | **0,00217** | **0,00217** | **0,00217** | **0,00217** | **0,00217** | **0,00217** | **0,00001** | **0,00142** |
| **D(4,3)** | **0,00000** | **0,00000** | **0,00000** | **0,00000** | **0,00000** | **0,00000** | **0,00000** | **0,00003** | **0,00000** | **0,00001** | **0,00000** | **0,00000** | **0,00002** | **0,00062** | **0,00003** |  | 0,68580 | **0,00000** | **0,00000** | **0,00264** | **0,00327** | **0,00376** | **0,00146** | **0,00019** | **0,00021** | **0,00245** | **0,00057** | **0,00000** | **0,00000** |
| **mode** | **0,00000** | **0,00000** | **0,00132** | **0,00000** | **0,00000** | **0,00000** | **0,00000** | **0,00083** | **0,00000** | 0,33890 | **0,00000** | **0,00000** | **0,00007** | 0,18480 | **0,00595** | 0,68580 |  | **0,00000** | **0,00000** | 0,54670 | 0,54670 | 0,54670 | 0,54670 | 0,31860 | 0,34040 | 0,54670 | 0,54670 | **0,00000** | **0,00000** |
| **span** | **0,00128** | **0,00000** | **0,00000** | **0,00000** | **0,00000** | **0,00000** | **0,00000** | **0,00350** | **0,00000** | **0,00000** | **0,00000** | **0,00000** | **0,00264** | **0,00002** | **0,00002** | **0,00000** | **0,00000** |  | **0,00000** | **0,00002** | **0,00002** | **0,00002** | **0,00002** | **0,00002** | **0,00002** | **0,00002** | **0,00002** | **0,00009** | **0,00000** |
| **surface area** | **0,00000** | **0,00001** | **0,00000** | **0,00000** | **0,00000** | **0,00000** | **0,00471** | **0,00002** | **0,00000** | **0,00000** | **0,00000** | **0,00000** | **0,00002** | **0,00002** | **0,00002** | **0,00000** | **0,00000** | **0,00000** |  | **0,00002** | **0,00002** | **0,00002** | **0,00002** | **0,00002** | **0,00002** | **0,00002** | **0,00002** | **0,00000** | 0,34940 |
| **FTIR 3280** | **0,00010** | **0,00011** | **0,00011** | **0,00010** | **0,00009** | **0,00010** | **0,00030** | **0,00217** | **0,00011** | **0,00016** | **0,00030** | **0,00580** | **0,00217** | 0,60930 | **0,00217** | **0,00264** | 0,54670 | **0,00002** | **0,00002** |  | 0,09670 | **0,00217** | **0,00729** | **0,00217** | **0,00217** | 0,60930 | **0,00217** | **0,00001** | **0,00142** |
| **FTIR 2926** | **0,00010** | **0,00011** | **0,00011** | **0,00010** | **0,00009** | **0,00010** | **0,00030** | **0,00217** | **0,00011** | **0,00030** | **0,00030** | **0,00580** | **0,00217** | 0,44330 | **0,00217** | **0,00327** | 0,54670 | **0,00002** | **0,00002** | 0,09670 |  | **0,00330** | **0,00330** | **0,00217** | **0,00217** | 0,07364 | **0,00217** | **0,00001** | **0,00142** |
| **FTIR 2853** | **0,00010** | **0,00011** | **0,00011** | **0,00010** | **0,00009** | **0,00010** | **0,00030** | **0,00217** | **0,00011** | **0,00485** | **0,00030** | **0,00580** | **0,00217** | 0,20130 | **0,00217** | **0,00376** | 0,54670 | **0,00002** | **0,00002** | **0,00217** | **0,00330** |  | **0,00217** | **0,00217** | **0,00217** | **0,00330** | **0,00217** | **0,00001** | **0,00142** |
| **FTIR 1648** | **0,00010** | **0,00011** | **0,00011** | **0,00010** | **0,00009** | **0,00010** | **0,00030** | **0,00217** | **0,00011** | **0,00010** | **0,00030** | **0,00297** | **0,00217** | 0,70150 | **0,00217** | **0,00146** | 0,54670 | **0,00002** | **0,00002** | **0,00729** | **0,00330** | **0,00217** |  | **0,00217** | **0,00217** | **0,00729** | **0,01060** | **0,00001** | **0,00142** |
| **FTIR 1537** | **0,00010** | **0,00011** | **0,00025** | **0,00010** | **0,00009** | **0,00010** | **0,00030** | **0,00217** | **0,00011** | **0,00010** | **0,00030** | **0,00011** | **0,00217** | 0,79830 | **0,00217** | **0,00019** | 0,31860 | **0,00002** | **0,00002** | **0,00217** | **0,00217** | **0,00217** | **0,00217** |  | 0,79830 | **0,00217** | 0,05528 | **0,00001** | **0,00142** |
| **FTIR 1408** | **0,00010** | **0,00011** | **0,00027** | **0,00010** | **0,00009** | **0,00010** | **0,00030** | **0,00217** | **0,00011** | **0,00010** | **0,00030** | **0,00011** | **0,00217** | 0,70150 | **0,00217** | **0,00021** | 0,34040 | **0,00002** | **0,00002** | **0,00217** | **0,00217** | **0,00217** | **0,00217** | 0,79830 |  | **0,00217** | 0,09670 | **0,00001** | **0,00142** |
| **FTIR 1234** | **0,00010** | **0,00011** | **0,00011** | **0,00010** | **0,00009** | **0,00010** | **0,00030** | **0,00217** | **0,00011** | **0,00020** | **0,00030** | **0,00580** | **0,00217** | 0,60930 | **0,00217** | **0,00245** | 0,54670 | **0,00002** | **0,00002** | 0,60930 | 0,07364 | **0,00330** | **0,00729** | **0,00217** | **0,00217** |  | **0,00217** | **0,00001** | **0,00142** |
| **FTIR 1050** | **0,00010** | **0,00011** | **0,00011** | **0,00010** | **0,00009** | **0,00010** | **0,00030** | **0,00217** | **0,00011** | **0,00010** | **0,00030** | **0,00020** | **0,00217** | 1,00000 | **0,00217** | **0,00057** | 0,54670 | **0,00002** | **0,00002** | **0,00217** | **0,00217** | **0,00217** | **0,01060** | 0,05528 | 0,09670 | **0,00217** |  | **0,00001** | **0,00142** |
| **Np** | 0,49400 | **0,00000** | **0,00000** | **0,00000** | **0,00000** | **0,00000** | **0,00000** | 0,10940 | **0,00000** | **0,00000** | 0,15310 | **0,00000** | 0,80870 | **0,00016** | **0,00001** | **0,00000** | **0,00000** | **0,00009** | **0,00000** | **0,00001** | **0,00001** | **0,00001** | **0,00001** | **0,00001** | **0,00001** | **0,00001** | **0,00001** |  | **0,00000** |
| **f (RPM)** | **0,00000** | **0,00000** | **0,00000** | **0,00000** | **0,00000** | **0,00000** | **0,00055** | **0,00100** | **0,00000** | **0,00000** | **0,00002** | **0,00000** | **0,00142** | **0,00142** | **0,00142** | **0,00000** | **0,00000** | **0,00000** | 0,34940 | **0,00142** | **0,00142** | **0,00142** | **0,00142** | **0,00142** | **0,00142** | **0,00142** | **0,00142** | **0,00000** |  |

Table S3: One-way PERMANOVA test for physico-chemical parameters with Bonferroni corrected p values for pretreated samples.

|  | R1-5 | R1-15 | R1-30 | R2-5 | R2-15 | R2-30 |
| --- | --- | --- | --- | --- | --- | --- |
| R1-5 |  | **0.0104** | 0.07 | **0.0002** | **0.0001** | **0.0003** |
| R1-15 | **0.0104** |  | **0.0051** | **0.0001** | **0.0001** | **0.0002** |
| R1-30 | 0.07 | **0.0051** |  | **0.0002** | **0.0003** | **0.0004** |
| R2-5 | **0.0002** | **0.0001** | **0.0002** |  | **0.0001** | **0.0006** |
| R2-15 | **0.0001** | **0.0001** | **0.0003** | **0.0001** |  | **0.0003** |
| R2-30 | **0.0003** | **0.0002** | **0.0004** | **0.0006** | **0.0003** |  |

Table S: One-way PERMANOVA test for physico-chemical parameters with Bonferroni corrected p values for untreated and pretreated samples.

| 0 | 0 | R1-5 | R1-15 | R1-30 | R2-5 | R2-15 | R2-30 |
| --- | --- | --- | --- | --- | --- | --- | --- |
| 0 |  | **0.0001** | **0.0001** | **0.0001** | **0.0002** | **0.0001** | **0.0003** |
| R1-5 | **0.0001** |  | **0.0075** | 0.0677 | **0.0001** | **0.0002** | **0.0003** |
| R1-15 | **0.0001** | **0.0075** |  | **0.0073** | **0.0002** | **0.0002** | **0.0004** |
| R1-30 | **0.0001** | 0.0677 | **0.0073** |  | **0.0001** | **0.0001** | **0.0002** |
| R2-5 | **0.0002** | **0.0001** | **0.0002** | **0.0001** |  | **0.0001** | **0.0005** |
| R2-15 | **0.0001** | **0.0002** | **0.0002** | **0.0001** | **0.0001** |  | **0.0002** |
| R2-30 | **0.0003** | **0.0003** | **0.0004** | **0.0002** | **0.0005** | **0.0002** |  |

**Figure S1.** Raw FTIR spectra of analyzed WAS samples

**Figure S2.** Raw FTIR spectra of analyzed WAS samples
